# Searching for new genes associated with the familial hypercholesterolemia phenotype using whole-genome sequencing and machine learning

**DOI:** 10.18699/VJGB-23-63

**Published:** 2023-09

**Authors:** D.E. Ivanoshchuk, A.B. Kolker, O.V. Timoshchenko, S.E. Semaev, E.V. Shakhtshneider

**Affiliations:** Institute of Internal and Preventive Medicine – Branch of the Institute of Cytology and Genetics of the Siberian Branch of the Russian Academy of Sciences, Novosibirsk, Russia Institute of Cytology and Genetics of the Siberian Branch of the Russian Academy of Sciences, Novosibirsk, Russia; Novosibirsk State Technical University, Novosibirsk, Russia; Institute of Internal and Preventive Medicine – Branch of the Institute of Cytology and Genetics of the Siberian Branch of the Russian Academy of Sciences, Novosibirsk, Russia; Institute of Internal and Preventive Medicine – Branch of the Institute of Cytology and Genetics of the Siberian Branch of the Russian Academy of Sciences, Novosibirsk, Russia Institute of Cytology and Genetics of the Siberian Branch of the Russian Academy of Sciences, Novosibirsk, Russia; Institute of Internal and Preventive Medicine – Branch of the Institute of Cytology and Genetics of the Siberian Branch of the Russian Academy of Sciences, Novosibirsk, Russia Institute of Cytology and Genetics of the Siberian Branch of the Russian Academy of Sciences, Novosibirsk, Russia

**Keywords:** familial hypercholesterolemia, whole-genome sequencing, machine learning, SIDT1, LRP1B, PLD1, CETP, семейная гиперхолестеринемия, полногеномное секвенирование, машинное обучение, SIDT1, LRP1B, PLD1, CETP

## Abstract

One of the most common congenital metabolic disorders is familial hypercholesterolemia. Familial hypercholesterolemia is a condition caused by a type of genetic defect leading to a decreased rate of removal of low-density lipoproteins from the bloodstream and a pronounced increase in the blood level of total cholesterol. This disease leads to the early development of cardiovascular diseases of atherosclerotic etiology. Familial hypercholesterolemia is a monogenic disease that is predominantly autosomal dominant. Rare pathogenic variants in the LDLR gene are present in 75–85 % of cases with an identified molecular genetic cause of the disease, and variants in other genes (APOB, PCSK9, LDLRAP1, ABCG5, ABCG8, and others) occur at a frequency of < 5 % in this group of patients. A negative result of genetic screening for pathogenic variants in genes of the low-density lipoprotein receptor and its ligands does not rule out a diagnosis of familial hypercholesterolemia. In 20–40 % of cases, molecular genetic testing fails to detect changes in the above genes. The aim of this work was to search for new genes associated with the familial hypercholesterolemia phenotype by modern high-tech methods of sequencing and machine learning. On the basis of a group of patients with familial hypercholesterolemia (enrolled according to the Dutch Lipid Clinic Network Criteria and including cases confirmed by molecular genetic analysis), decision trees were constructed, which made it possible to identify cases in the study population that require additional molecular genetic analysis. Five probands were identified as having the severest familial hypercholesterolemia without pathogenic variants in the studied genes and were analyzed by whole-genome sequencing on the HiSeq 1500 platform (Illumina). The whole-genome sequencing revealed rare variants in three out of five analyzed patients: a heterozygous variant (rs760657350) located in a splicing acceptor site in the PLD1 gene (c.2430-1G>A), a previously undescribed single-nucleotide deletion in the SIDT1 gene [c.2426del (p.Leu809CysfsTer2)], new missense variant c.10313C>G (p.Pro3438Arg) in the LRP1B gene, and single-nucleotide deletion variant rs753876598 [c.165del (p.Ser56AlafsTer11)] in the CETP gene. All these variants were found for the first time in patients with a clinical diagnosis of familial hypercholesterolemia. Variants were identified that may influence the formation of the familial hypercholesterolemia phenotype.

## Abstract (ru)

Одним из наиболее распространенных врожденных метаболических нарушений является семейная гиперхолестеринемия. Это заболевание приводит к раннему развитию сердечно-сосудистых заболеваний атеросклеротического генеза. Семейная гиперхолестеринемия относится к моногенным заболеваниям с преимущественно аутосомно-доминантным типом наследования. Редкие патогенные варианты в гене LDLR определяются в 75–85 % случаев у пациентов с выявленной молекулярно-генетической причиной заболевания, варианты в других генах встречаются с частотой менее 5 % (APOB, PCSK9, LDLRAP1, ABCG5, ABCG8 и др.). Отрицательный результат генетического скрининга патогенных вариантов генов рецептора липопротеинов низкой плотности и его лигандов не исключает диагноз «семейная гиперхолестеринемия». В 20–40 % случаев при моле- кулярно-генетическом исследовании не удается определить изменения в вышеназванных генах. Цель настоящей работы – поиск новых генов, ассоциированных с фенотипом семейной гиперхолестеринемии, с использованием современных высокотехнологичных методов секвенирования и машинного обучения. На основании выборки пациентов с семейной гиперхолестеринемией, сформированной по критериям Dutch Lipid Clinic Network и включающей случаи заболевания, подтвержденные молекулярно-генетическим анализом, построены решающие деревья, которые позволили выделить из выборки случаи, требующие дополнительного молекулярно-генетического анализа. Определены пять пробандов с наиболее тяжелым течением семейной гиперхолестеринемии без патогенных вариантов в изученных генах для проведения полногеномного секвенирования на платформе HiSeq 1500 (Illumina). При выполнении полногеномного секвенирования у трех из пяти обследо ванных пациентов найдены редкие варианты: гетерозиготный вариант (rs760657350), локализованный в акцепторном сайте сплайсинга гена PLD1: c.2430-1G>A, ранее не описанная однонуклеотидная делеция гена SIDT1: c.2426del (p.Leu809CysfsTer2), новый миссенс-вариант c.10313C>G (p.Pro3438Arg) гена LRP1B и вариант однонуклеотидной делеции rs753876598: c.165del (p.Ser56AlafsTer11) гена CETP. Все варианты впервые описаны у пробандов с клиническим диагнозом «семейная гиперхолестеринемия». Идентифицированы варианты, которые потенциально могут влиять на формирование фенотипа семейной гиперхолестеринемии.

## Introduction

One of the most common congenital metabolic disorders is familial hypercholesterolemia (FH) (Ezhov et al., 2019). Familial hypercholesterolemia is a condition caused by a type of genetic defects leading to a decreased rate of removal of low-density lipoproteins from the bloodstream and a pronounced increase in the blood level of total cholesterol (Ezhov et al., 2019). This illness leads to early development of cardiovascular diseases of atherosclerotic origin (Wiegman et al., 2015; Santos et al., 2016; Borén et al., 2020). Familial hypercholesterolemia is a monogenic disease with predominantly autosomal dominant inheritance (Ezhov et al., 2019).

The prevalence of the heterozygous type of FH in white populations is 1 per 250 people (Ezhov et al., 2019). Rare pathogenic variants in the LDLR gene are present in 75–85 % of cases with an identified molecular genetic cause of the disease, and variants in other genes (APOB, PCSK9, LDLRAP1, ABCG5, ABCG8, and others) occur in this group of patients with a frequency of less than 5 % (Nordestgaard et al., 2013; Iacocca, Hegele, 2017; Vasilyev et al., 2020). Patients can be homozygous or heterozygous carriers of pathogenic variants, and this status determines the severity of the disease and onset age of manifestations of cardiovascular complications (Vaezi, Amini, 2022). A negative result of genetic screening for pathogenic variants does not rule out familial hypercholesterolemia. In 20–40 % of cases, changes in the above genes are absent according to molecular genetic analysis. The risk of coronary heart disease among patients with FH is 20 times higher in the absence of treatment (Khera et al., 2016); therefore, it is important and relevant to search for new approaches to identifying patients at early disease stages and to assessing predisposition to this disease in patients’ families

To search for new cases, some authors have proposed a classifier to identify potential patients with FH by means of electronic medical records. Using data from patients with confirmed FH (n = 197) and cases without FH (n = 6590), a decision tree classifier was trained in that study. The classifier showed a positive predictive value (PPV) of 0.88 and a sensitivity of 0.75 for long-term testing. This classifier proved to be effective at finding candidate patients for further screening for familial hypercholesterolemia. Such machine-learning-based strategies can result in efficient identification of patients having the highest risk of the disease (Banda et al., 2019).

For the diagnosis of FH, clinicians use the principle of cascade genetic screening. The latter is a step-by-step identification of patients with familial hypercholesterolemia. When elevated blood levels of total cholesterol and low-density lipoprotein cholesterol (LDL-C) are detected in a patient, his/her family history of health problems is collected and clinical manifestations are analyzed. In case of a diagnosis of “probable” or “definite” FH according to the Dutch Lipid Clinic Network Criteria (Geneva: World Health Organization), the patient is referred for molecular genetic testing. The cascade screening includes quantitation of blood lipids in all first-degree relatives of the proband. If the FH diagnosis is confirmed by the molecular genetic methods, then genetic screening is performed on his/her relatives. As new patients with FH are identified, their relatives are examined too. The cascade screening is the most effective way to detect previously undiagnosed FH (Nordestgaard et al., 2013; Ezhov et al., 2019)

The aim of the present work was to search for new genes associated with the FH phenotype using modern high-tech methods of sequencing and machine learning.

## Materials and methods

A group of patients with FH (ICD-10 Е78.0, Е78.2, n = 102), was recruited from a clinical diagnostic department at the Institute of Internal and Preventive Medicine (IIPM) – a branch of the Institute of Cytology and Genetics, the Siberian Branch of the Russian Academy of Sciences (ICG SB RAS). The study protocol was approved by the Ethics Committee at the IIPM – a branch of the ICG SB RAS, decision No. 68 of June 4, 2019. Informed consent was obtained from each study participant.

The diagnosis of FH was made in accordance with the clinical lipid criteria of the Dutch Lipid Clinic Network (DLCN) (WHO-Human genetics DoNDP…, 1999). According to the criteria, a score was computed (see the Supplementary)1 for patients with familial hypercholesterolemia. The patients underwent a medical examination, ultrasonographic diagnostics, and blood sampling for biochemical assays (lipid profiling and general analysis of biochemical parameters) and molecular genetic assays.

Supplementary Materials are available in the online version of the paper: http://vavilov.elpub.ru/jour/manager/files/Suppl_Ivanoshchuk_Engl_27_5.pdf


Blood samples for the biochemical analyses were collected once from the cubital vein in the morning on an empty stomach at 12 h after a meal. Serum levels of total cholesterol, triglycerides, LDL-C, high-density lipoprotein cholesterol (HDL-C), and glucose were determined by enzymatic methods on a KoneLab300i automatic biochemical analyzer (Finland) with reagents from TermoFisher (Finland). The LDL-C level was calculated via the Friedewald formula; at the LDL-C concentration of > 4.5 mmol/L, the method of direct quantification of LDL-C was used. Statistical analysis of the data was performed in the SPSS software for Windows, version 23.0.

Phenol-chloroform extraction was carried out to isolate DNA from blood (Sambrook, Russell, 2006). The quality of the extracted DNA was assessed with the help of an Agilent 2100 Bioanalyzer capillary electrophoresis system (Agilent Technologies Inc., USA).

Targeted DNA sequencing in patients with FH was performed on the MiSeq platform (Illumina) using a customdesigned panel of 43 genes: LDLR, APOB, PCSK9, LDLRAP1, CETP, LPL, HMGCR, NPC1L1, PPARA, MTTP, LMF1, SAR1B, ABCA1, ABCG5, ABCG8, CYP7A1, STAP1, LIPA, PNPLA5, APOA1, APOA5, APOC2, APOE, LCAT, ANGPTL3, LIPC, APOA4, APOC3, SREBF1, LMNA, PPARG, PLIN1, POLD1, LPA, SMAD1, SMAD2, SMAD3, SMAD4, SMAD5, SMAD6, SMAD7, SMAD9, and LIPG (NimbleGen SeqCap Target Enrichment, Roche, Switzerland).

At the next stage of this work, from the study population, 42 patients with FH were chosen who did not show pathogenic variants in the tested genes during the targeted sequencing analysis. These patients were subjected to multiplex ligationdependent probe amplification (MLPA) analysis to identify possible sequence alterations (deletions or duplications) in the LDLR gene promoter and exons by means of SALSA MLPA KIT P062 (MRCHolland, Amsterdam, the Netherlands).

Using the group of patients with FH (compiled according to the DLCN criteria and including cases of the disease confirmed by molecular genetic analysis), decision trees were constructed, which enabled us to identify cases in this group that require additional molecular genetic analysis. Software was written in Python 3.9 for building a set of decision rules for predicting FH on the basis of machine learning with a limited training set. The decision rules were stored as data representation in the Predictive Model Markup Language. The decision rules were built by means of a labeled database of patients with a diagnosis of FH (Certificate of Database Registration: RU No. 2023660511; software registration application of May 2, 2023).

By machine learning methods, five probands with the severest FH without pathogenic variants in the tested genes were identified for subsequent whole-genome sequencing on the HiSeq 1500 platform (Illumina). Automated processing and annotation of the obtained sequencing data were conducted on the NGS Wizard platform (genomenal.ru). The sequence reads were mapped to the reference human genome (GRCh38/ hg38). A potential effect of novel missense variants on protein function/structure was assessed using data from in silico prediction tools (CADD (https://cadd.gs.washington.edu/snv), PolyPhen2 (http://genetics.bwh.harvard.edu/pph2/), and MutationTaster (https://www.mutationtaster.org/)) and data from gnomAD on the frequency of these variants in populations. In this way, variants (in genes associated with lipid metabolism) leading to a loss of protein function and missense variants with a frequency of less than 0.01 % were selected. Pathogenicity of new variants was evaluated according to the guidelines of the American College of Medical Genetics and Genomics (ACMG) and the Association for Molecular Pathology (Richards et al., 2015). Analysis of protein-protein interaction networks was performed in STRING (Szklarczyk et al., 2019).

## Results

By targeted sequencing and MLPA, “pathogenic” and “likely pathogenic” variants were detected in 47.5 % of the examined probands and in 85.7 % of the probands’ children. Variants in the LDLR gene that were identified in patients with the FH phenotype in our study are presented in Table 1. All missense variants were found to be in a heterozygous state. Variants Cys352Tyr, Cys340Phe, and Leu401His have been previously described in patients with FH in Russia (Zakharova et al., 2005) and in other countries (Feussner et al., 1996; Torres et al., 2014).

**Table 1.**
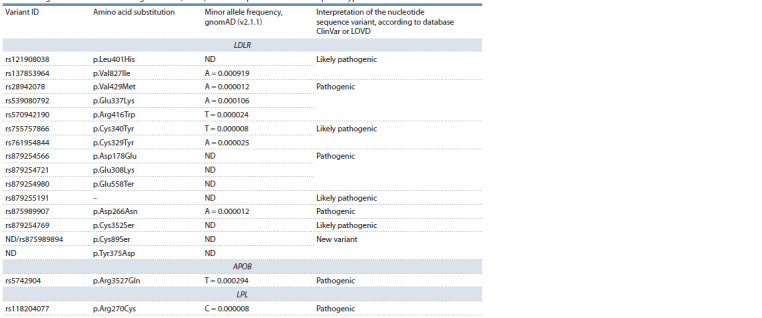
Single-nucleotide variants in genes LDLR, APOB, and LPL of patients with the FH phenotype ND – no data; GenBank accession numbers of protein sequences that were used in variant annotation: LDLR (NP_000518.1), APOB (NP_000375.3), and LPL (NP_000228.1).

In the study population, the heterozygous type of the disease was due to rare variants in the LDLR gene in 73 % of the cases. Two new variants – NM_000527.5:c.266G>C, NP_000518.1:p.Cys89Ser and NM_000527.5:c.1123T>G, NP_000518.1:p.Tyr375Asp – were identified in the LDLR gene. Two unrelated probands turned out to be carriers of compound heterozygous variants of the LDLR gene, whereas the clinical course of the disease in these patients corresponded to the homozygous type of familial hypercholesterolemia. In the first case, in a 28-year-old female patient with a diagnosis of definite FH, we detected rare variants NM_000527.5:c.796G>A, NP_000518.1:p.Asp266Asn, and NM_000527.5:c.1054T>A, NP_000518.1:p.Cys352Ser in exons 5 and 7 of LDLR (see Table 1). In the second case, a 35-year-old female patient with a diagnosis of definite FH has two missense variants in exons 3 and 8 of LDLR. One substitution is located in LDLR exon 3 (NM_000527.5:c.266G>C, NP_000518.1:p.Cys89Ser), in which at this position, rare likely pathogenic variant rs875989894 NM_000527.5:c.266G>A, NP_000518.1:p.Cys89Tyr has been previously described in patients with FH (Day et al., 1997; Graham et al., 1999; Fouchier et al., 2005).

The pathogenicity of the identified variant was confirmed by in silico analysis (Mutation Taster score: 112, CADD score: 24.8, PolyPhen-2 score: 1.000). The other new missense variant is situated in exon 8 of LDLR: NM_000527.5:c.1123T>G, NP_000518.1:p.Tyr375Asp (see Table 1). This missense variant causes an amino acid substitution at the same position where other missense variants have been described before as likely pathogenic in patients with FH (Assouline et al., 1997; García-García et al., 2001; Damgaard et al., 2005; Mollaki et al., 2014). The pathogenicity of the variant was also confirmed by in silico analysis (Mutation Taster score: 160, CADD score: 25.5, PolyPhen-2 score: 1.000). Both detected variants (NM_000527.5:c.266G>C, NP_000518.1:p.Cys89Ser and NM_000527.5:c.1123T>G, NP_000518.1:p.Tyr375Asp) are not annotated in the gnomAD database (v2.1.1). According to this evidence, both were assumed to be likely pathogenic variants.

Patients without functionally significant substitutions in lipid metabolism genes were analyzed by MLPA to determine sequence changes (deletions or duplications) in the promoter and exons of the LDLR gene. This assay revealed a deletion of a coding region in the LDLR gene [NM_000527.4:c. (2140+1_2141-1)_(2311+1_2312-1)del] in DNA samples from two unrelated patients.

In the molecular genetic analysis, in three patients from two unrelated families (a proband and a son of the proband from one family and a proband from another family), we identified variant rs5742904 (NM_000384.3:c.10580G>A, NP_000375.3:p.Arg3527Gln) (ClinVar Variation ID:17890) in the APOB gene (see Table 1).

Rare substitutions in the APOB gene region encoding the LDL receptor-inding site are associated with hypercholesterolemia. One of the variants in this region, NP_000375.3:p. Arg3527Gln, leads to hypercholesterolemia with reduced clearance of LDL-C owing to a defect in the structural motif of LDL that is responsible for affinity for LDL receptor (Pullinger et al., 1995).

Analysis of our data of targeted high-throughput sequencing revealed rare pathogenic variant rs118204077 (NM_000237.3:c.808C>T, NP_000228.1:p.Arg270Cys) in the LPL gene in a heterozygous state (ClinVar Variation ID: 1548) (see Table 1). This variant was found in a 45-year-old male patient with hypercholesterolemia (12.4 mmol/L) and hypertriglyceridemia (17.4 mmol/L; DLCN score: 5). Earlier, variants associated with hypertriglyceridemia have been described at this locus (Ma et al., 1994; Surendran et al., 2012) in patients with lipoprotein lipase deficiency (Hegele et al., 2018; Teramoto et al., 2018).

According to the results of the molecular genetic analyses, 52.5 % of our patients are not carriers of pathogenic variants in the studied lipid metabolism genes. Among these patients, by a machine learning algorithm, five subjects with the severest FH were chosen for whole-genome sequencing. As a result, in three patients, four variants with a minor allele frequency of less than 0.01 % were identified in genes related to lipid metabolism. Among these variants, two are single-nucleotide deletions, one affects a splicing acceptor site, and one is a missense variant. The findings are presented in Table 2.

**Table 2.**
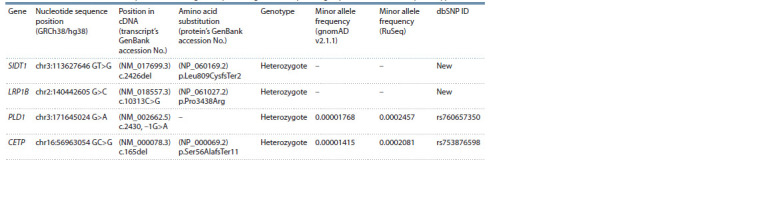
Rare variants identified in lipid metabolism genes by whole-genome sequencing in patients with the FH phenotype

In the SIDT1 gene (encoding a protein called SID1 transmembrane family member 1), a new previously undescribed variant was detected that yields a frameshift starting with codon 809 (NM_017699.3:c.2426de, NP_060169.2:p. Leu809CysfsTer2). The gene consists of 30 exons and is located in chromosomal region 3q13.2 (https://www.ncbi.nlm. nih.gov/gene/54847). According to gnomAD, loss-of-function variants in this gene have been documented, but according to ClinVar and the literature, none of them have been annotated as pathogenic or likely pathogenic. For this gene, the pLI score was found to be 0, indicating that the gene is resistant to lossof- function variants. The weight of evidence suggested that this variant has uncertain clinical significance (pathogenicity criterion PM2)

A new missense variant was found in the LRP1B gene (LDL receptor-related protein 1B): c.10313C>G p.Pro3438Arg in a heterozygous state. The gene is located in chromosomal region 2q22.1-q22.2, consists of 92 exons, and encodes one of the receptors of the LDL receptor family (https://www.ncbi.nlm. nih.gov/gene/53353). In gnomAD, there are no data on the frequency of this variant. The pathogenicity of this variant was also corroborated by in silico analysis (Mutation Taster score: 103, CADD score: 33, PolyPhen-2 score: 1.000). Most variants in this gene either are benign (data from ClinVar, accessed in February 2023) or have uncertain clinical significance. The totality of the data indicated that this variant has uncertain clinical significance (pathogenicity criteria PM2, PP3, and BP1).

One of our patients proved to be a carrier of a rare heterozygous variant at a splicing acceptor site (NM_002662.5:c.2430, –1G>A) in the phospholipase D1 (PLD1) gene. This variant is registered in a control sample in gnomAD: five mutant alleles on 282,768 chromosomes (no homozygotes have been detected). The PLD1 gene codes for a phosphatidylcholinespecific phospholipase that catalyzes the hydrolysis of phosphatidylcholine, thus yielding phosphatidic acid and choline (https://www.ncbi.nlm.nih.gov/gene/5337). The gene is situated in chromosomal region 3q26.31 and contains 35 exons. Phospholipase D (PLD) and its enzymatic reaction product, phosphatidic acid, regulate adhesion of immune cells (macrophages and neutrophils) to collagen (Speranza et al., 2014). It is known that biallelic variants with loss of function of the PLD1 gene cause neonatal cardiomyopathy and congenital malformations of the pulmonary valve and tricuspid valve, of the right ventricle of the heart, and of the outflow tract of the right ventricle (Ta-Shma et al., 2017; Lahrouchi et al., 2021). The weight of evidence suggested that this substitution is a likely pathogenic variant in relation to congenital heart malformations (pathogenicity criteria PM2 and PVS1). With respect to FH, we categorized the detected substitution as a variant of unknown clinical significance (pathogenicity criterion PM2).

One of the examined patients was found to have a heterozygous single-nucleotide deletion in CETP: rs753876598 (NM_000078.3:c.165del) (https://www.ncbi.nlm.nih.gov/ gene/1071). The variant is annotated in the ClinVar database (ID1675625) and is registered in a control sample of gnomAD: four mutant alleles on 282,774 chromosomes (no homozygotes have been found). It is known that variants causing loss of function of this gene affect the HDL-C level (Millwood et al., 2018; Li et al., 2021). According to the totality of criteria for pathogenicity evaluation (PM2 and PVS1), we designated this variant as likely pathogenic. The CETP gene codes for a plasma protein that catalyzes the exchange of triglycerides and cholesterol esters between lipoprotein particles (Oliveira, Raposo, 2020).

## Discussion

High-throughput sequencing is employed not only for molecular genetic diagnosis of FH but also as a tool for identifying i) variants that may be involved in lipid metabolism and ii) their effects on the phenotype of patients with FH (Miroshni- kova et al., 2021). In the current study, 16 variants were identified in an FH population (15 single-nucleotide substitu- tions and one deletion) that have previously been classified as pathogenic or likely pathogenic in the ClinVar or LOVD database as well as two new missense variants in the LDLR gene that we classified as pathogenic. In our genome-wide analysis, in lipid metabolism-associated genes, we detected four additional variants that met our search criteria. Two of these four variants have been described before, and two are new.

One of the genes in which rare variants were found in patients with FH is PLD1, encoding phospholipase D1. This enzyme hydrolyzes membrane lipid phosphatidylcholine thereby generating phosphatidic acid (Bowling et al., 2021). Phosphatidic acid is an intermediate metabolite in the synthesis of all membrane glycerophospholipids and plays an important structural role in live cells by promoting membrane biogenesis (Tanguy et al., 2018); furthermore, its involvement and phospholipase D1’s participation in exocytosis have been demonstrated (Tanguy et al., 2022).

Alternative splicing of PLD1 mRNA results in many different transcripts having both catalytic and regulatory functions (Nelson, Frohman, 2015). It has been shown that recessive variants in the PLD1 gene are associated with severe right-sided congenital heart malformations in two families (Ta-Shma et al., 2017). In Pld1 knockout mice, moderate dysfunction of pulmonary and tricuspid valves is observed (Ta-Shma et al., 2017). Recessive PLD1 variants also correlate with isolated neonatal cardiomyopathies (Lahrouchi et al., 2021). In humans, missense variants of PLD1 are reported to be concentrated in regions of the protein critical for catalytic activity, thus resulting in low enzymatic activity in most of such mutant proteins (Lahrouchi et al., 2021). It has also been demonstrated in cell lines that PLD1 overexpression promotes the formation of lipid droplets, whereas an siRNA PLD1 knockdown inhibits this process (Andersson et al., 2006).

The variant (NM_002662.5:c.2430, –1G>A) that we detected in a proband with FH without signs of congenital heart disease is in a heterozygous state. Considering the low prevalence of this variant and its possible role in subcellular transport and in the formation of lipid droplets, this substitution is of interest for further investigation in individuals with lipid metabolism disorders.

Another rare variant was found by us in the LRP1B gene (LDL receptor-related protein 1B). The LRP1B protein is a member of the LDL receptor family (Strickland et al., 2002). LRP1B takes part in lipoprotein catabolism; accordingly, research on rare variants of the LRP1B gene in individuals with FH is promising. Most of the recently identified ligands of LRP1B are well-known factors of blood coagulation and of lipoprotein metabolism, suggesting that LRP1B is implicated in atherosclerosis (Lee, 2019).

SIDT1 is a multispan transmembrane protein belonging to the SID1 transmembrane family and shares some sequence homology with Caenorhabditis elegans ChUP-1, which is a cholesterol-binding protein located in intracellular vesicles (Valdes et al., 2012). SIDT1 expression in endolysosomes has been documented (Nguyen et al., 2019). SIDT1 has been shown to participate in cholesterol transport (Méndez-Acevedo et al., 2017) but has not been investigated in the context of the FH phenotype. Most likely, the variant that we found in this gene does not take part in the formation of the clinical phenotype of FH because our assessment using the American College of Medical Genetics and Genomics criteria classifies it as a variant of uncertain clinical significance; however, for unambiguous evaluation of its association with the FH phenotype, additional data are needed.

The CETP gene codes for the CETP protein, which carries cholesterol esters. This protein regulates the concentration and particle size of HDL-C in the blood and plays an important role in reverse cholesterol transport (Barter, Kastelein, 2006). It has been shown that elevated activity of CETP reduces HDL-C concentration and correlates with a higher risk of cardiovascular disease (Barter, 2011; Iwanicka et al., 2018). Variants in the CETP gene can alter the blood lipid profile (Wuni et al., 2022). In our previous study on one of the CETP variants, we reported its association with changes in the blood lipid profile and with the risk of myocardial infarction in a population of Western Siberia (Semaev et al., 2019). When a map of functional and physical associations was constructed in the present study, the APOB protein turned out to be a predicted functional partner of the CETP protein, and mutations in APOB represent some of FH etiologies.

Additional segregational and functional analyses are necessary to evaluate pathogenic effects of the identified variants on the formation of the clinical FH phenotype. Identification of new pathogenic variants will facilitate risk assessment of FH and of its complications among patients and members of their families.

## Conclusion

A combination of machine learning and whole-genome sequencing in probands with a clinical diagnosis of FH revealed rare variants in genes SIDT1, LRP1B, PLD1, and CETP; these variants may influence the disease phenotype.

## Conflict of interest

The authors declare no conflict of interest.
